# Safety, Tolerability, and Pharmacokinetics of Anti-SARS-CoV-2 Immunoglobulin Intravenous (Human) Investigational Product (COVID-HIGIV) in Healthy Adults: a Randomized, Controlled, Double-Blinded, Phase 1 Study

**DOI:** 10.1128/aac.01514-22

**Published:** 2023-02-28

**Authors:** Sean T. H. Liu, Mila Mirceta, Grace Lin, Deborah M. Anderson, Tarashon Broomes, Alina Jen, Ashley Abid, David Reich, Christine Hall, Judith A. Aberg

**Affiliations:** a Division of Infectious Diseases, Department of Medicine, Icahn School of Medicine at Mount Sinai, New York, New York, USA; b Emergent BioSolutions Canada, Inc., Winnipeg, Manitoba, Canada

**Keywords:** COVID-19, hyperimmune immunoglobulin, antiviral pharmacology

## Abstract

Anti-SARS-CoV-2 immunoglobulin (human) investigational product (COVID-HIGIV) is a purified immunoglobulin preparation containing SARS-CoV-2 polyclonal antibodies. This single-center clinical trial aimed to characterize the safety and pharmacokinetics of COVID-HIGIV in healthy, adult volunteers. Participants were enrolled to receive one of three doses of COVID-HIGIV (100, 200, 400 mg/kg) or placebo in a 2:2:2:1 randomization scheme. Between 24 December 2020 and 27 July 2021, 28 participants met eligibility and were randomized with 27 of these 28 (96.4%) being administered either COVID-HIGIV (*n* = 23) or placebo (*n* = 4). Only one SAE was observed, and it occurred in the placebo group. A total of 18 out of 27 participants (66.7%) reported 50 adverse events (AEs) overall. All COVID-HIGIV-related adverse events were mild or moderate in severity and transient. The most frequent AEs (>5% of participants) reported in the safety population were headache (*n* = 6, 22.2%), chills (*n* = 3, 11.1%), increased bilirubin (*n* = 2, 7.4%), muscle spasms (*n* = 2, 7.4%), seasonal allergies (*n* = 2, 7.4%), pyrexia (*n* = 2, 7.4%), and oropharyngeal pain (*n* = 2, 7.4%). Using the SARS-CoV-2 binding IgG immunoassay (*n* = 22, specific for pharmacokinetics), the geometric means of Cmax (AU/mL) for the three COVID-HIGIV dose levels (low to high) were 7.69, 17.02, and 33.27 AU/mL; the average values of T_max_ were 7.09, 7.93, and 5.36 h, respectively. The half-life of COVID-HIGIV per dose level was 24 d (583 h), 31 d (753 h), and 26 d (619 h) for the 100 mg/kg, 200 mg/kg, and 400 mg/kg groups, respectively. The safety and pharmacokinetics of COVID-HIGIV support its development as a single-dose regimen for postexposure prophylaxis or treatment of COVID-19.

## INTRODUCTION

Severe acute respiratory syndrome coronavirus 2 (SARS-CoV-2) is a novel coronavirus that emerged in December 2019 resulting in the coronavirus disease 2019 (COVID-19) pandemic. Several authorized monoclonal antibody (MAb) cocktails have demonstrated reduced efficacy against certain emerging SARS-CoV-2 variants (1 to 3). Following the emergence of the Omicron variant and its subvariants, casirivimab plus imdevimab and bamlanivimab plus etesevimab MAb cocktails, as well as sotrovimab, have had their emergency use authorizations (EUAs) revised to not be authorized for use in U.S. regions where nonsusceptible variants are dominant (4 to 6). Recently, polyclonal convalescent plasma has been shown to reduce the relative risk of progression of disease to hospitalization when administered early after the onset of symptoms (within 9 days) in recipients who were mostly unvaccinated (7). Although COVID-19 convalescent plasma (CCP) and anti-SARS-CoV-2 hyperimmune immunoglobulin (COVID-HIGIV) have not demonstrated treatment efficacy in hospitalized patients (8 to 16), polyclonal antibodies are typically more robust and resistant to mutations and variants as they bind a diverse array of epitopes (17, 18). The lack of efficacy in many of these studies of patients with severe COVID-19 seems to be mostly influenced by (i) the variability of antibody titers from plasma donors, with most studies having low titers, and (ii) delayed timing of treatment post-symptom onset, when there is likely to be reduced benefit later in the disease course. Despite this apparent lack of efficacy in hospitalized patients, CCP has been associated with greater viral clearance than standard of care (14, 15), and improved outcomes with early treatment using high-titer plasma (19), suggesting that clearing the viral infection in late-stage COVID-19 patients is not sufficient to improve outcomes; it is likely host inflammatory dysregulation that is influencing disease progression in late-stage patients. A polyclonal hyperimmune product, like COVID-HIGIV, presents as an option for a highly potent form of variant-resistant, passive immunotherapy to reduce the disease burden and improve clinical outcomes early in disease onset.

This study evaluated the safety and pharmacokinetics (PK) of three COVID-HIGIV dose levels administered intravenously as a single dose to healthy adults. COVID-HIGIV is similar to other human immunoglobulin intravenous (IGIV) products indicated for varicella-zoster, hepatitis B, anthrax, or vaccinia (20 to 24). We searched PubMed for research articles published between database inception and 19 April 2022, using various combinations of the terms “COVID-19,” “SARS-CoV-2,” “human hyperimmune immunoglobulin,” “pharmacokinetics,” and “clinical trial.” No language or date restrictions were applied. Review articles, nonhuman hyperimmune products, case series, and articles describing hyperimmune production processes were omitted. Preclinical studies in hamsters challenged with SARS-CoV-2 given a single dose of COVID-HIGIV (400 mg/kg) showed significant reductions in viral replication (25). COVID-HIGIV from vaccinated donors neutralized a wide variety of SARS-CoV-2 variants, including D614G, Alpha (B.1.1.7), Beta (B.1.351), Gamma (P.1), Kappa (B.1.617.1), Delta (B.1.617.2), and Omicron (B.1.1.529) *in vitro* (17). Beyond neutralization, IgG Fc-dependent pathways may play a role in combatting SARS-CoV-2 infections using COVID-HIGIV (26). The ITAC (INSIGHT 013) Study Group did not demonstrate treatment efficacy of COVID-HIGIV among patients hospitalized with COVID-19 without end-organ failure (13). Additional *in vitro* data suggest that COVID-HIGIV could be used for preexposure and postexposure prophylaxis of high-risk individuals against currently circulating SARS-CoV-2 Delta and Omicron variants compared with convalescent plasma or prepandemic IGIV (18). Results from the current report provided below offer safety information on COVID-HIGIV, as well as PK parameters such as peak plasma levels, trough titers of SARS-CoV-2 antibodies, and half-life in healthy adults. These data may inform dose optimization/dose selection for the proposed indications of treatment of COVID-19 and postexposure prophylaxis of COVID-19. 

## RESULTS

### Recruitment.

**FIG 1:**
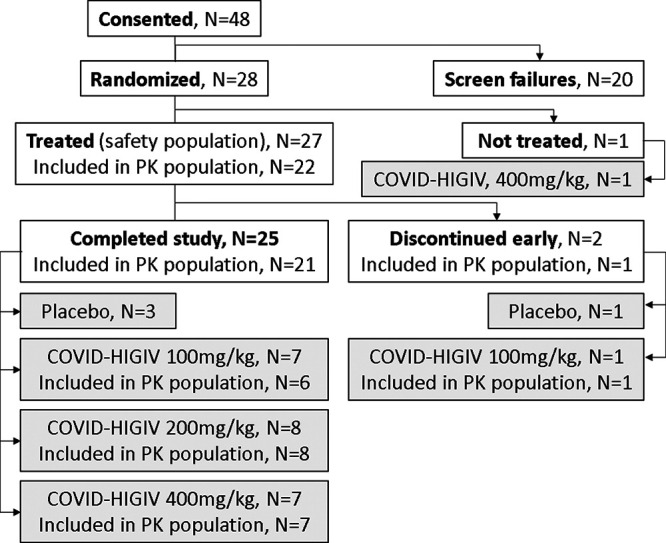
Disposition flow chart of participants for study EBS-CVH-003.

Between 24 December 2020 and 27 July 2021, 48 individuals were assessed for eligibility; a total of 28 individuals met the study eligibility criteria and were randomized, with 27 of these 28 (96.4%) being administered either COVID-HIGIV (*n* = 23) or placebo (*n* = 4) (Fig. 1). One participant randomized to the COVID-HIGIV 400 mg/kg arm did not receive investigational product due to a predose adverse event of vasovagal syncope (Fig. 1, “Not treated”).

### Baseline data.

Of the 27 participants in the safety population, 17 (63.0%) were males and 10 (37.0%) were females, with a mean age of 30.6 years and baseline body max index of 25.3 kg/m2 (Table 1). Approximately half of the participants were White (51.9%), with Black or African American, Other, and Asian representing 25.9%, 18.5%, and 3.7% of participants, respectively.

**TABLE 1:** Summary of participant demographics at baseline (safety population)^*a*^

Parameter	COVID-HIGIV 100 mg/kg (*N* = 8)	COVID-HIGIV 200 mg/kg (*N* = 8)	COVID-HIGIV 400 mg/kg (*N* = 7)	Placebo (*N* = 4)	Total (*N* = 27)
Sex at birth *n* (%)					
Male	5 (62.5)	5 (62.5)	4 (57.1)	3 (75.0)	17 (63.0)
Female	3 (37.5)	3 (37.5)	3 (42.9)	1 (25.0)	10 (37.0)
Age (yrs)					
Mean (SD)	28.4 (10.2)	31.5 (9.5)	33.9 (14.0)	27.8 (8.3)	30.6 (10.5)
Median	25	29.5	28	26.5	26
Min, Max	19, 50	21, 49	23, 56	19, 39	19, 56
Race *n* (%)					
Asian	1 (12.5)	0	0 (0)	0 (0)	1 (3.7)
Black or African American	2 (25.0)	2 (25.0)	2 (28.6)	1 (25.0)	7 (25.9)
Other	0	1 (12.5)	2 (28.6)	2 (50.0)	5 (18.5)
White	5 (62.5)	5 (62.5)	3 (42.9)	1 (25.0)	14 (51.9)
Ethnicity *n* (%)					
Not Hispanic or Latino	7 (87.5)	6 (75.0)	4 (57.1)	2 (50.0)	19 (70.4)
Hispanic or Latino	1 (12.5)	2 (25.0)	3 (42.9)	2 (50.0)	8 (29.6)
Ht (m)					
Mean (SD)	173.0 (8.9)	172.1 (12.2)	168.4 (12.3)	184.8 (14.9)	173.3 (12.2)
Median	171.5	171.5	170.2	185.4	170.2
Min, Max	162.6, 193.0	154.9, 190.5	149.9, 185.4	167.6, 200.7	149.9, 200.7
Baseline wt (kg)					
Mean (SD)	72.2 (11.9)	79.3 (12.4)	74.1 (15.9)	80.8 (15.7)	76.1 (13.4)
Median	70.1	81.9	79.6	81.9	79.6
Min, Max	57.2, 90.3	54.0, 92.5	55.3, 91.6	64.0, 95.3	54.0, 95.3
Baseline body mass index (kg/m^2^)					
Mean (SD)	24.1 (3.7)	26.9 (4.5)	25.9 (3.2)	23.5 (1.2)	25.3 (3.7)
Median	23.6	27.7	24.6	23.3	24.3
Min, Max	19.2, 32.1	19.8, 32.9	21.1, 31.0	22.4, 25.0	19.2, 32.9

a SD, standard deviation; Min, minimum; Max, maximum; kg, kilogram; *N*, number of participants in safety set by study arm; *n*, number of participants in specified category; %, *n*/N*100.

### Numbers analyzed.

The analysis populations in this study consisted of the safety population (*n* = 27) and the PK population (*n* = 22). The safety population included all participants who received any amount of study drug (COVID-HIGIV or placebo). The enrolled safety population totaled 27 participants, including *n* = 8 for COVID-HIGIV at 100 mg/kg; *n* = 8 for COVID-HIGIV at 200 mg/kg; *n* = 7 for COVID-HIGIV at 400 mg/kg; and *n* = 4 for placebo. Three out of four participants in the placebo cohort completed the study, with one participant experiencing an unrelated serious adverse event (SAE) on day 28, requesting emergency unblinding and subsequently being lost to follow-up. The PK population included all participants who received COVID-HIGIV and had an adequate set of evaluable PK samples (a suitable predose sample and at least one measurable postdose sample). Twenty-two out of 23 dosed participants (95.7%) in the COVID-HIGIV groups completed the study, with one participant receiving 100 mg/kg of COVID-HIGIV lost to follow-up. Included in the 22 participants in the PK population were *n* = 7 for COVID-HIGIV at 100 mg/kg; *n* = 8 for COVID-HIGIV at 200 mg/kg; and *n* = 7 for COVID-HIGIV at 400 mg/kg (Table 2). One participant in the COVID-HIGIV 100 mg/kg group was excluded from the T_1/2_ calculation due to extremely high predose and postdose PK values (>ULOQ [upper limit of quantification]).

**TABLE 2:** Summary of SARS-CoV-2 S-protein binding IgG antibody PK parameters (PK population)^*a*^

PK parameter	Unit	Statistic	COVID-HIGIV 100 mg/kg *N* = 7	COVID-HIGIV 200 mg/kg *N* = 8	COVID-HIGIV 400 mg/kg *N* = 7
C_max_	AU/mL	*n*	7	8	7
		Mean (SD)	7.79 (1.32)	17.10 (1.81)	33.63 (5.26)
		Median	7.30	16.65	33.30
		Min, Max	6.4, 9.6	14.8, 20.7	26.1, 39.5
		Geo. Mean	7.69	17.02	33.27
		Geo. Mean 95% CI	6.60, 8.97	15.63, 18.54	28.69, 38.58
		Geo. CV%	16.71	10.23	16.11
T_max_	h	n	7	8	7
		Mean (SD)	7.09 (7.84)	7.93 (6.81)	5.36 (1.46)
		Median	4.40	6.15	4.40
		Min, Max	3.2, 24.7	3.3, 24.4	3.9, 7.5
		Geo. Mean	5.23	6.48	5.19
		Geo. Mean 95% CI	2.68, 10.20	3.90, 10.78	4.06, 6.64
		Geo. CV%	82.88	66.96	27.11
AUC_0-last_	h*AU/mL	*n*	7	8	7
		Mean (SD)	3075.21 (891.12)	6724.25 (827.61)	11853.30 (1917.45)
		Median	3277.20	6833.50	11013.30
		Min, Max	1966.9, 4224.1	5589.8, 7823.7	9541.0, 14642.0
		Geo. Mean	2961.93	6678.80	11722.82
		Geo. Mean 95% CI	2246.17, 3905.77	6014.80, 7416.11	10108.67, 13594.72
		Geo. CV%	30.59	12.57	16.12
T_1/2_	h	*n*	6	8	7
		Mean (SD)	583.13 (237.39)	752.87 (257.67)	618.79 (148.64)
		Median	521.45	659.55	625.60
		Min, Max	344.7, 921.7	445.8, 1281.0	363.8, 812.0
		Geo. Mean	544.73	718.74	601.56
		Geo. Mean 95% CI	356.91, 831.39	550.17, 938.95	470.26, 769.53
		Geo. CV%	41.98	32.80	27.10

a d, day; Geo CV%, coefficient of variation; Geo. Mean, geometric mean; h, hour; *N*, number of participants in PK set by study arm; *n*, number of participants with evaluable data; PK, pharmacokinetic.

### Outcomes and estimation.

**(i) Analysis of adverse events.** A total of 18 out of 27 participants (66.7%) reported 50 AEs overall. Of the 18 participants with AEs, 15 were from the COVID-HIGIV groups compared to 3 participants in the placebo group. There were 29 out of 50 AEs (58%) in 15 out of 27 participants (55.5%) that were considered treatment-related by the investigator. A total of 26 AEs in 13 (56.5%) participants were reported to be related to COVID-HIGIV compared to three AEs in two (50%) participants considered related to placebo (Table 3). There was a dose-dependent relationship in the frequency of related AEs, with 6 of the 7 (85.7%) participants in the highest dose of COVID-HIGIV (400 mg/kg) experiencing 14 AEs. The most frequent COVID-HIGIV-related AE was headache in five (21.7%) participants, with four of these participants receiving 400 mg/kg COVID-HIGIV. Blurred vision and dehydration occurred in one participant during the infusion, prompting a temporary suspension. Chills and muscle spasms occurred in two (8.7%) participants across all three of the COVID-HIGIV-treated groups. The most frequent AEs (>5% of participants) reported in the safety population were headache (22.2%), chills (11.1%), increased bilirubin (7.4%), muscle spasms (7.4%), seasonal allergies (7.4%), pyrexia (7.4%), and oropharyngeal pain (7.4%).

**TABLE 3:** Treatment-emergent adverse events assessed as related to study drug by MedDRA system organ class and preferred term (safety population)^*a*^

System organ class preferred term	COVID-HIGIV 100 mg/kg (*N* = 8) *n* (%)	COVID-HIGIV 200 mg/kg (*N* = 8) *n* (%)	COVID-HIGIV 400 mg/kg (*N* = 7) *n* (%)	Placebo (*N* = 4) *n* (%)
No. participants with a related TEAE	3 (37.5)	4 (50.0)	6 (85.7)	2 (50.0)
Blood and lymphatic system disorders	0 (0)	0 (0)	1 (14.3)	0 (0)
Anemia	0 (0)	0 (0)	1 (14.3)	0 (0)
Eye disorders	0 (0)	1 (12.5)	0 (0)	0 (0)
Vision blurred	0 (0)	1 (12.5)	0 (0)	0 (0)
Gastrointestinal disorders	0 (0)	1 (12.5)	0 (0)	1 (25.0)
Abdominal pain	0 (0)	1 (12.5)	0 (0)	0 (0)
Frequent bowel movements	0 (0)	0 (0)	0 (0)	1 (25.0)
Nausea	0 (0)	1 (12.5)	0 (0)	0 (0)
General disorders and administration site conditions	1 (12.5)	1 (12.5)	1 (14.3)	1 (25.0)
Chills	1 (12.5)	0 (0)	1 (14.3)	0 (0)
Fatigue	0 (0)	0 (0)	0 (0)	1 (25.0)
Infusion site pain	0 (0)	1 (12.5)	0 (0)	0 (0)
Pyrexia	0 (0)	0 (0)	1 (14.3)	0 (0)
Investigations	1 (12.5)	0 (0)	2 (28.6)	1 (25.0)
Alanine aminotransferase increased	0 (0)	0 (0)	1 (14.3)	0 (0)
Blood bilirubin increased	1 (12.5)	0 (0)	0 (0)	1 (25.0)
Blood lactate dehydrogenase increased	0 (0)	0 (0)	1 (14.3)	0 (0)
Metabolism and nutrition disorders	0 (0)	1 (12.5)	0 (0)	0 (0)
Dehydration	0 (0)	1 (12.5)	0 (0)	0 (0)
Musculoskeletal and connective tissue disorders	1 (12.5)	1 (12.5)	1 (14.3)	0 (0)
Back pain	0 (0)	1 (12.5)	0 (0)	0 (0)
Muscle spasms	1 (12.5)	0 (0)	1 (14.3)	0 (0)
Myalgia	0 (0)	0 (0)	1 (14.3)	0 (0)
Nervous system disorders	1 (12.5)	0 (0)	4 (57.1)	0 (0)
Headache	1 (12.5)	0 (0)	4 (57.1)	0 (0)
Somnolence	0 (0)	0 (0)	1 (14.3)	0 (0)
Renal and urinary disorders	0 (0)	0 (0)	1 (14.3)	0 (0)
Haematuria	0 (0)	0 (0)	1 (14.3)	0 (0)
Skin and subcutaneous tissue disorders	0 (0)	1 (12.5)	0 (0)	0 (0)
Pruritus	0 (0)	1 (12.5)	0 (0)	0 (0)

a *N*, number of participants in safety set by study arm; *n*, number of participants in specified category; TEAE, treatment-emergent adverse event; %, *n*/N*100. Adverse events are coded to MedDRA version 23.0. Participants with more than one event are counted once per participant, system organ class (SOC), and preferred term (PT) with the maximum toxicity grade. SOC and PT are sorted in alphabetical order.

Eleven participants received medication due to AEs, with four of these being due to AEs that were assessed as related to study treatment. Paracetamol was the concomitant medication given for all four of these related AEs, which were caused by headaches of either grade 1 (1 participant) or grade 2 (3 participants) severity that all resolved within the same day. All four of the participants that required paracetamol received the highest dose of COVID-HIGIV (400 mg/kg). One serious adverse event (SAE) not related to study treatment occurred in the placebo group on study day 28 of study follow-up. No safety signal stopping rules were met. No participants tested positive for COVID-19 over the course of the study follow-up.

Eleven participants had out-of-range laboratory assessments postdosing, including 9 COVID-HIGIV treated and 2 placebo-treated. Sixteen of these out-of-range clinical laboratory results had a toxicity grade of 1 and four had a toxicity grade of 2. Eleven of these occurred between screening and the day 4 assessment. Two participants in the placebo and 100 mg/kg COVID-HIGIV groups, respectively, had clinically significant elevated bilirubin levels on day 2 that were assessed as possibly related AEs by the investigator. Two other possibly related AEs, elevated alanine aminotransferase and asymptomatic hematuria assessed through urinalysis, were observed in two participants in the 400 mg/kg COVID-HIGIV group postdosing on day 2. No other abnormal laboratory results were considered clinically significant. Overall, the COVID-HIGIV product appeared safe and well-tolerated in this population of healthy adult participants.

**(ii) Analysis of pharmacokinetics.** Pharmacokinetic analyses were performed based on the PK population using the results from the validated SARS-CoV-2 binding IgG immunoassay targeting anti-SARS-CoV-2 antibodies that bind the SARS-CoV-2 S-protein trimer. Serum samples collected after COVID-19 infection or vaccination were excluded from PK analyses. This occurred for one participant in the 100 mg/kg group and one participant in the 200 mg/kg group, who were vaccinated after the day 57 visit but prior to day 85. Day 85 data were excluded for these two participants for PK analyses. An additional participant in the 100 mg/kg group had serum antibody levels that were greater than ULOQ at baseline, indicating either prior infection or vaccination that was not detected during screening. This participant was excluded from the PK analysis population.

**FIG 2:**
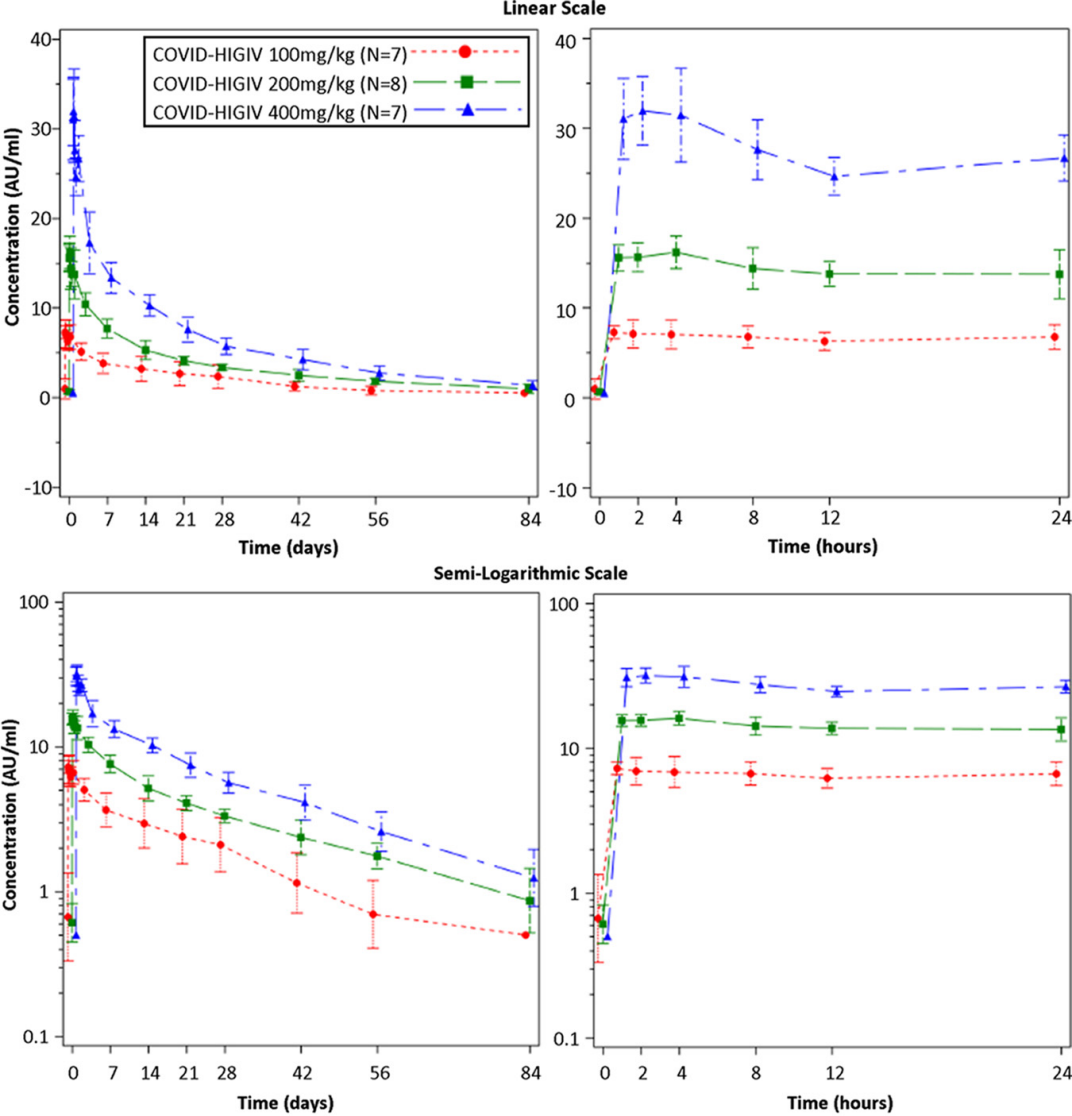
Mean (±SD) concentration-time profiles for COVID-HIGIV S-protein binding antibodies on linear scale (upper panels) and semilogarithmic scale (lower panels) over 84 days (left) and 24 h (right) postdose (PK population).

Using the SARS-CoV-2 binding IgG immunoassay, the geometric means of Cmax (AU/mL) for the three COVID-HIGIV dose levels (low to high) were 7.69, 17.02, and 33.27 AU/mL; the average values of T_max_ were 7.09, 7.93, and 5.36 h, respectively (Table 2). The geometric means of AUC_0-last_ (h*AU/mL) were 2962, 6679, and 11723, respectively; the geometric means of minimum concentration through day 29 (Cmin28d) were 2.06, 3.33, and 5.47 AU/mL, respectively. As expected, there was a dose-dependent increase in various PK parameters. The half-life of COVID-HIGIV per dose level was 24 d (583 h), 31 d (753 h), and 26 d (619 h) for the 100 mg/kg, 200 mg/kg, and 400 mg/kg groups, respectively. Fig. 2 illustrates the mean serum concentration of anti-SARS-CoV-2 S-protein binding antibodies over time per dose level of COVID-HIGIV up to day 84 and up to 24 h postdose, shown linearly and semilogarithmically.

## DISCUSSION

Study EBS-CVH-003 was a phase 1 single-center, double-blind, randomized, placebo-controlled study to assess the safety and pharmacokinetics of COVID-HIGIV following intravenous administration in healthy adult participants. The most frequent AEs (>5% of participants) reported in the safety population were headache (22.2%), chills (11.1%), increased bilirubin (7.4%), muscle spasms (7.4%), seasonal allergies (7.4%), pyrexia (7.4%), and oropharyngeal pain (7.4%). Of these common AEs, headache, increased bilirubin, and muscle spasms were considered related to COVID-HIGIV by the investigator at the time of the event. AEs related to musculoskeletal and connective tissue disorders (arthralgia, back pain, muscle spasms, and myalgia) were frequently reported in the COVID-HIGIV groups (5 participants; 21.7% of those receiving COVID-HIGIV), but none was observed in placebo participants. These frequently observed AEs were expected for a human IGIV product. AEs were more commonly reported for the highest COVID-HIGIV dose level (400 mg/kg). Most related AEs were of mild intensity and self-limited (observed in 9 of 13 participants experiencing related AEs; 69.2%), indicative of an overall favorable safety profile for COVID-HIGIV.

The PK results for COVID-HIGIV obtained in this study were similar to the PK of other human IGIV products manufactured by Emergent and other commercial IGIV products (20 to 24). As demonstrated in this study, COVID-HIGIV had a T_max_ of 5.36 to 7.09 h and a calculated mean half-life between 24 to 31 days (based on the binding IgG immunoassay) across the three dose levels, which is in the range of the expected half-life for this product class. Vaccinia Immune Globulin Intravenous (VIGIV) is one example, where the T_max_ is 1.84 to 2.61 h and the calculated mean half-life is between 26.2 to 30.0 days (24). Similarly, anthrax immunoglobulin (human) injection has a T_max_ of 2.78 to 4.06 h and a calculated mean half-life between 24.3 to 28.0 days (23). The C_max_ and area under the concentration-time curve (AUC) of COVID-HIGIV are difficult to compare with those of other human-derived IgG products as unit measurements of concentration vary from assay to assay depending on the product being evaluated. Dose proportionality assessed via the power model revealed model-predicted slopes above the upper limit of the prespecified proportionality criterion (90% confidence interval [0.9, 1.1]), suggesting nonlinear pharmacokinetics for COVID-HIGIV. The minimum concentration through day 29 (C_min28d_) was lower than expected, indicating that dosing may need to be increased for future studies that would assess maintenance dosing.

A limitation of this study was that the pseudoviral neutralizing antibody assay was not suitable for evaluating PK parameters for COVID-HIGIV due to its reduced sensitivity to antibody concentrations below 60 AU/mL. Such a high lower limit of quantitation (LLOQ) brings into question the utility of this assay for future evaluations of serum-neutralizing antibody concentrations. Further assay optimization would be necessary to be able to quantify PK parameters of neutralizing S-protein antibodies at lower concentrations in participants who are SARS-CoV-2 antibody negative at baseline. Future studies with COVID-HIGIV in participants that are antibody positive at baseline may be a feasible approach to generate PK data for SARS-CoV-2 neutralizing antibodies using this assay, as there would likely not be a limitation due to the LLOQ. Another limitation of the study was that mean age of the participants was 30.6 years of age with a range of 19 to 56 years. This may not be the target patient population if COVID-HIGIV will be used as a treatment for persons at high risk for severe disease. The high-risk population is typically greater than 65 years old.

COVID-HIGIV is a sterile liquid preparation of the purified IgG fraction of human plasma containing polyclonal antibodies to SARS-CoV-2. COVID-HIGIV is being developed for postexposure prophylaxis and treatment of COVID-19. Given the reduced efficacy of some MAbs against emerging variants (1 to 3), development of a polyclonal antibody therapeutic is critical to ensuring that effective antibody treatments are available long-term.

The safety of human immunoglobulins is well established, and the most common types of adverse reactions to IGIV and hyperimmune products are nonanaphylactic infusion reactions (self-limiting), such as abdominal or back pain, fever, headache, chills, rash, fatigue, nausea, or vomiting. The incidence of adverse reactions associated with IGIVs is in the range of 1 to 15%, typically ≤5% (20 to 24, 27 to 29). Infrequent events associated with IGIV administration (i.e., product class-specific) have also been reported, such as hypersensitivity reactions, renal dysfunction/failure, aseptic meningitis syndrome, hemolysis, transfusion-related acute lung injury, and thrombotic events. There were no such events reported in the 23 participants who were administered COVID-HIGIV in this study. Most of the AEs related to COVID-HIGIV were considered mild and self-limited. Overall, COVID-HIGIV appeared safe and well-tolerated in healthy adult participants based on the lack of clinically significant safety observations, and had a PK profile consistent with the expected PK characteristics of other commercially available human hyperimmune products. Future considerations for research include study of COVID-HIGIV with higher SARS-CoV-2 neutralizing activity, to allow for lower dose volumes and other routes of administration.

## MATERIALS AND METHODS

### Trial design.

This study was a phase 1, single-center, randomized, double-blind, placebo-controlled design to assess the safety and PK of COVID-HIGIV in healthy adults. Eligible participants were randomized in a ratio of 2:2:2:1 to one of four study treatment arms to receive a single intravenous (IV) infusion of one of three COVID-HIGIV dose levels (arms 1 to 3) or saline placebo (arm 4), respectively. Assessment and sampling time points included predose (baseline) and postdose at 1 h, 2 h, 4 h, 8 h, 12 h, day 2, day 4, day 8, day 15, day 22, day 29, day 43, day 57, and day 85 or at the withdrawal visit.

Noteworthy protocol modifications were made in each protocol amendment. Importantly, urine drug screening and a history of elective surgery were removed from exclusion criteria as they would not definitively lead to protocol noncompliance. Guidelines pertaining to COVID-19 vaccination during study conduct were revised so that those who received placebo could receive the COVID-19 vaccine.

### Participants.

Healthy nonpregnant adults aged 18 to 60 years were eligible for inclusion (see supplemental materials for full eligibility criteria). Participants were negative for spike (S) protein antibodies at screening and had no known history of prior exposure to SARS-CoV-2 virus or vaccination. The study was done at Mount Sinai Hospital (New York City, New York, USA) following approval by Advarra IRB (no. Pro00046735). All participants gave written informed consent before enrollment. The study is registered with ClinicalTrials.gov (NCT04661839).

### Study product: COVID-HIGIV.

COVID-HIGIV is prepared from pooled plasma collected at U.S. FDA-licensed and/or registered plasma and/or blood collection centers from healthy, adult donors who have elevated levels of anti-SARS-CoV-2 antibodies. COVID-HIGIV activity against SARS-CoV-2 (product potency) was determined by a validated binding IgG immunoassay (458 AU/mL) that measures the concentration of binding SARS-CoV-2 antibodies (with SARS-CoV-2 trimeric S protein as the antigen) as well as a pseudoviral neutralization assay (3121 AU/mL) that is a validated lentivirus-based SARS-CoV-2 S-protein pseudotype neutralization assay (expressed in Alliance Units [AU]/mL) in relation to a reference standard (purified IgG prepared from pooled plasma with anti-SARS-CoV-2 binding and neutralizing activity). The final drug product is also characterized by a wild-type SARS-CoV-2 neutralization assay (816 AU/mL) performed by NIAID (30). The calculated potency of the COVID-HIGIV clinical lot used in this study (finished good lot 23003552) using each of these three assays is provided above. For preparation of COVID-HIGIV, a sterile syringe and standard aseptic technique were used to transfer the appropriate volume to an appropriately sized and labeled IV bag. IV bags were diluted with normal saline up to a total volume of 400 mL.

### Interventions.

Study participants were randomized to receive one of three dose levels as a single 400-mL dose volume of either COVID-HIGIV (*n* = 24) or placebo control (*n* = 4) administered by IV infusion. Participants were admitted to the phase 1 hospital-based unit on the day they were intended to receive their dose of investigational product (day 1). Baseline assessments were done prior to randomization. COVID-HIGIV was administered by IV infusion through a dedicated IV line using a constant infusion pump. The starting infusion rate was 1.0 mL/min for the first 30 min. If the infusion was well tolerated, the infusion rate was doubled to 2.0 mL/min for the next 30 min, and if tolerated, the rate was doubled to 4.0 mL/min (maximum rate) for the remainder of the infusion. If AEs occurred during the IV infusion, the rate of infusion may have been slowed or temporarily stopped until symptoms subsided at the discretion of the investigator. Participants were typically dosed in the morning to early afternoon on day 1. Participants remained in the unit until assessments within the first 24 h postdosing were completed (i.e., participants were discharged on day 2).

### Outcomes.

The primary outcomes were the nature, incidence, and severity of adverse events, as well as the incidence of clinically significant changes and abnormalities in safety laboratory parameters (hematology, coagulation, biochemistry, and urinalysis), and vital signs (body temperature, blood pressure, heart rate, and respiratory rate). Monitoring for COVID-19 cases and symptoms was included in the safety analyses. The medical assessment of adverse event severity was done in accordance with the DAIDS Table for Grading the Severity of Adult and Pediatric Adverse Events (mild = grade 1, moderate = grade 2, severe = grade 3, potentially life-threatening = grade 4).

The following primary PK endpoints (based on PK sample test results of a binding IgG immunoassay and a pseudoviral neutralization assay) were evaluated: AUC_0-last_: area under the concentration-time curve (AUC) from time zero to the last quantifiable concentration after dosing; AUC_0-inf_: AUC_0-last_ plus the additional area extrapolated to infinity after dosing; AUC_0-14d_ after dosing; AUC_0-28d_ after dosing; C_max_: maximum observed concentration after dosing; T_max_: time at which C_max_ occurs after dosing; C_min28d_: observed or estimated concentration at 28 days after dosing; λ_z_: terminal elimination rate constant after dosing; T_1/2_: apparent terminal elimination half-life after dosing; CL: systemic clearance after dosing; VZ: volume of distribution after dosing.

No major changes were made to the planned analyses. Due to pseudoviral neutralization assay sensitivity issues, where the serum concentrations for most samples were below LLOQ for this assay (60 AU/mL), there were limited PK results from the pseudoviral neutralization assay, with only the COVID-HIGIV 400 mg/kg arm having sufficient serum concentration data up to day 4 before dropping below the LLOQ. As such, these data were deemed inappropriate and excluded in this report. The observed concentration at 28 days after dosing (C_min28d_) was reported for binding IgG immunoassay alone, without interpolation or extrapolation.

### Sample size.

There was no formal sample size calculation for this study. However, the number of participants planned to receive COVID-HIGIV was deemed sufficient to descriptively assess safety and PK of three COVID-HIGIV dose levels of healthy adults. In total, 28 participants were planned for the study: eight participants for each of the three COVID-HIGIV dose levels, and four participants for the placebo arm.

### Randomization.

Randomization schedule generation and logistics were handled by an unblinded contract research organization (CRO) statistician (Axiom Real-Time Metrics, Inc., Etobicoke, Ontario, Canada) who was not involved in study treatment administration or participant assessments.

### Blinding.

Participants and investigators were unaware of treatment allocation in this study. Investigators enrolled participants. The pharmacy staff was provided unblinded access to the randomization assignment to prepare the study treatment. To maintain the blind, both the study drug and placebo had the same administration volume (400.0 mL), and study treatments were prepared in IV infusion bags that were covered with protective coverings to mask any differences between COVID-HIGIV and placebo appearance during IV infusion. Designated personnel at the sponsor’s bioanalytical laboratory assigned to perform PK sample testing were indirectly unblinded due to the nature of the test results, but measures were implemented not to disseminate this information until after database lock. The PK statistician remained blinded to subject COVID-HIGIV dose level throughout the study.

Due to rapidly changing guidelines around the COVID-19 pandemic, a protocol amendment allowed participants to be unblinded in the study if they were eligible to receive the COVID-19 vaccine. Based on the Advisory Committee on Immunization Practices (ACIP) vaccination guidelines at the time, stating that those receiving passive immunotherapy should wait 90 days posttreatment to get vaccinated, only those that were in the placebo group were able to get vaccinated prior to study completion.

### Statistical methods.

All derivations, statistical analyses, summaries, and listings were generated using SAS version 9.4 or higher (SAS Institute, Inc., Cary, North Carolina, United States). There were two analysis populations, the safety population and the PK population. The safety population included all randomized participants who received any amount of study treatment (COVID-HIGIV or placebo). All safety analyses were based on the safety population. The PK population included all randomized participants who received any amount of study treatment (COVID-HIGIV) according to the protocol and had an adequate set of evaluable PK samples (a suitable predose sample and at least one measurable postdose sample). A validated binding IgG immunoassay targeting anti-SARS-CoV-2 antibodies was used for the principal PK analyses. Serum concentration versus time data was analyzed by standard noncompartmental methods (i.e., linear trapezoidal method for consecutive time points with level or increasing concentrations and log-linear trapezoidal method for consecutive time points with decreasing concentrations) done manually using SAS software. Actual sample collection times and not nominal times were used in PK parameter estimates.
