# Supplementary material for: Safety, Tolerability, and Pharmacokinetics of Anti-SARS-CoV-2 Immunoglobulin Intravenous (Human) Investigational Product (COVID-HIGIV) in Healthy Adults: a Randomized, Controlled, Double-Blinded, Phase 1 Study
Source: Antimicrob Agents Chemother. 2023 Feb 28;67(3):e01514-22. doi: 10.1128/aac.01514-22 (PMC10019156; doi:10.1128/aac.01514-22)
Supplement: Supplemental file 1 — Supplemental material. Download aac.01514-22-s0001.pdf, PDF file, 0.5 MB [file aac.01514-22-s0001.pdf]

**Supplemental materials**

**Selection of study population**

All participants enrolled in this study were healthy male and non-pregnant female volunteers who met the criteria outlined below.

**Inclusion criteria**

Participants met the following inclusion criteria to participate in the study:

1. Able and willing to provide written informed consent (voluntarily signed by the participant) prior to performing any study procedures.
2. Females or males, 18-60 years of age, inclusive.
3. Body mass index (BMI) less than or equal to 35.0 kg/m2.
4. Women who were either:

   A. Not of childbearing potential: surgically sterile (at least six weeks post bilateral tubal ligation, bilateral oophorectomy or hysterectomy), or post-menopausal (defined as ≥50 years of age with a history of ≥12 months without menses prior to randomization in the absence of other pathologic or physiologic causes, following cessation of exogenous sex-hormonal treatment); OR

   B. Women of childbearing potential (WOCBP) who were not planning to be pregnant during the study period and met all of the following criteria:

      i. Negative pregnancy test prior to randomization/dosing at Day 1; **and** Use of a highly effective contraception during the study period:

         - Hormonal contraceptives (e.g., implants, pills, patches) initiated ≥30 days prior to Day 1; or
         - Intrauterine device (IUD) inserted ≥30 days prior to Day 1; or
         - Double barrier type of birth control (e.g., male condom with female diaphragm, male condom with cervical cap).

5. Participant understood and agreed to comply with planned study procedures.
6. Healthy as determined by principal investigator based on medical history, physical exam, vital signs, urinalysis, blood chemistry and hematology test results at Screening and no evidence of prior exposure to SARS-CoV-2 (i.e., RT-PCR negative for SARS-CoV-2 and negative for SARS-CoV-2 antibodies) at Screening.

**Exclusion criteria**

Participants who had any of the following exclusion criteria were excluded from the study:

1. Use of any investigational product within 30 days prior to Screening or use of any SARS-CoV-2 vaccines or monoclonal antibodies, or COVID-19 convalescent plasma at any time prior to Screening or during study follow-up period, or participant planned to participate in another clinical study during the study period.

2. Screening clinical laboratory test result greater than the laboratory's upper limit of normal (ULN) for alanine aminotransferase (ALT), aspartate aminotransferase (AST), random glucose, total and/or bilirubin, blood urea nitrogen (BUN), or creatinine. Other serum chemistry parameters that are not within the reference range will not be considered exclusionary unless deemed clinically significant by the principal investigator.
3. History of hypersensitivity to blood or plasma products or to COVID-HIGIV excipients (proline, PS80).
4. History of allergy to latex or rubber.
5. History of hemolytic anemia.
6. History of IgA deficiency.
7. Receipt of any blood product within the past 12 months.
8. Plasma donation within 7 days or significant blood loss or blood donation within 56 days of randomization/dosing.
9. History of known congenital or acquired immunodeficiency or receipt of immunosuppressive therapy (e.g., prednisone or equivalent for more than two consecutive weeks within the past three months).
10. History of thrombosis or hypercoagulable state with increased risk of thrombosis.
11. History of clinically significant chronic illness (e.g., requiring hospitalization in the past three months) such as cardiac, pulmonary, renal, hepatic, or other chronic conditions.
12. Receipt of a live vaccine within 28 days prior to screening or anticipated receipt of a live vaccine during the study period.
13. Currently pregnant, breastfeeding, or planning to become pregnant during the study.
14. History of, or suspected substance abuse problem (including alcohol).
15. Other medical condition which might have placed the participant at increased risk due to participation in the study as determined by the investigator.
16. Any planned elective surgery or procedure during the follow-up period that impacts study compliance.
17. An opinion of the investigator that it would be unwise to allow the individual to be randomized into the study.
